# Progress and Challenges in Measles and Rubella Elimination in the WHO European Region

**DOI:** 10.3390/vaccines12060696

**Published:** 2024-06-20

**Authors:** Mark Muscat, Myriam Ben Mamou, Catharina Reynen-de Kat, Dragan Jankovic, José Hagan, Simarjit Singh, Siddhartha Sankar Datta

**Affiliations:** Vaccine-Preventable Diseases and Immunization Programme, World Health Organization Regional Office for Europe, DK-2100 Copenhagen, Denmark; benmamoum@who.int (M.B.M.); reynendekatc@who.int (C.R.-d.K.); jankovicd@who.int (D.J.); haganj@who.int (J.H.); singhsi@who.int (S.S.); dattas@who.int (S.S.D.)

**Keywords:** disease elimination, disease outbreaks, epidemiology, European region, measles, public health surveillance, rubella, vaccines, verification

## Abstract

The elimination of both measles and rubella remains a priority for all 53 Member States of the World Health Organization (WHO) European Region. To provide an update on the epidemiological status of measles and rubella in the Region, we reviewed surveillance data on both diseases for 2023 submitted monthly by national surveillance institutions. We analyzed the cases of measles and rubella for 2023 by age group, case classification, vaccination, hospitalization, and importation status and report on measles-related deaths. In 2023, 60,860 measles cases, including 13 fatal cases, were reported in 41 countries. Most cases (95%; n = 57,584) were reported by six countries: Azerbaijan, Kazakhstan, Kyrgyzstan, Romania, the Russian Federation, and Türkiye. Of the 60,848 cases with data on age, 19,137 (31%) were 1–4 years old and 12,838 (21%) were 5–9 years old. A total of 10,412 (17%) were 20 years and older. The genotypes identified in the Region were largely dominated by D8 variants (n = 1357) and the remainder were B3 variants (n = 221). In 2023, 345 rubella cases were reported by 17 countries, mostly from Poland, Kyrgyzstan, Tajikistan, Türkiye, and Ukraine. A total of 262 cases (76%) were classified as clinically compatible and 79 (23%) were laboratory-confirmed. To achieve the elimination of measles and rubella in the Region, political commitment needs to be revived to enable urgent efforts to increase vaccination coverage, improve surveillance and outbreak preparedness, and respond immediately to outbreaks.

## 1. Introduction

Measles and rubella incidence in the World Health Organization (WHO) European Region declined significantly in recent decades because of the widespread use and increasing coverage of measles- and rubella-containing vaccines (MRCVs) [1]. This success enabled its 53 Member States, all of which include two doses of MRCVs in their routine immunization schedules to jointly establish the ambitious goal of eliminating endemic measles and rubella transmission from the entire Region. In this context, disease elimination is defined as the interruption of endemic measles or rubella transmission in a defined geographical area such as a country or WHO Region for a period of at least 12 months in the presence of a well-performing surveillance system.

The Health for All policy framework for the WHO European Region (Health21), approved by the WHO Regional Committee for Europe in 1998, identified targets for nine vaccine-preventable diseases, including measles elimination by 2007 and the incidence of congenital rubella syndrome (CRS) of <1 case per 100,000 live births by 2010 [2]. The strategic plan for measles and congenital rubella infection in the WHO European Region targeted both the interruption of the endemic transmission of measles and the prevention of congenital rubella infection by 2010 [3]. In 2005, the inclusion of rubella elimination into the strategy was approved at the fifty-fifth session of the WHO Regional Committee for Europe as part of the resolution on strengthening national immunization systems through the elimination of measles and rubella and the prevention of congenital rubella infection [4]. In 2010, all of the Region’s 53 Member States reaffirmed their commitment to achieving this goal [5], which was subsequently included as a priority in the European Vaccine Action Plan 2015–2020 (EVAP) [6,7] and is an important component of the European Immunization Agenda 2030 (EIA2030) [8].

In this article, we provide an update on the epidemiological status of measles and rubella in the Region based on a review of surveillance data on both diseases for 2023. We also discussed the challenges and actions needed to reach the elimination goal for both diseases.

## 2. Materials and Methods

We present an analysis of preliminary measles and rubella surveillance data for 2023 and report on the number of cases of these diseases in the Region for 2013–2023 and by country for 2022. All surveillance data are as of 6 March 2024. We report on vaccination coverage with the first and second doses of MRCV (MRCV1 and MRCV2, respectively) for 2013 up to 2022, the last year for which coverage data were available at the time of submission of this article. We also report on measles and rubella virus sequence data as submitted by the national and regional reference laboratories of the WHO European Region for 2023 in the Measles Nucleotide Surveillance database (MeaNS) [9] and Rubella Nucleotide Surveillance database (RubeNS) [10], respectively, by 29 April 2024.

Surveillance data were submitted monthly by national surveillance institutions and incorporated in the WHO Immunization Information System (WIISE) [11]. Data from the 27 Member States of the European Union, Iceland, and Norway were entered into WIISE after collection and processing by The European Surveillance System—TESSy, of the European Centre for Disease Prevention and Control [12]. MRCV1 and MRCV2 coverage data were obtained from WHO/United Nations Children’s Fund Estimates of National Immunization Coverage (WUENIC) (as of 26 June 2023) [1]. We refer to the elimination status of measles and rubella as of 2022 as concluded by the European Regional Verification Commission for Measles and Rubella Elimination (RVC) at its most recent meeting held in 2023 [13].

The analyses of the surveillance data were performed on the cases with disease onset dates during 2022 and 2023. Where these dates were unavailable, the cases with the date of notification reported during these years, respectively, were included. The reported cases of measles and rubella for 2023 were analyzed by age group, case classification (laboratory-confirmed, epidemiologically linked, and clinically compatible [14]), vaccination, and importation status. The cases were categorized by age groups: younger than 1 year, 1–4 years, 5–9 years, 10–14 years, 15–19 years, 20–29 years, and 30 years and older. In addition, we report on hospitalization and measles-related deaths among the cases that occurred in 2023.

The incidence of disease was calculated with the number of cases of reported measles as the numerator and the country population obtained from the Population Division of the United Nations Organization [15] as the denominator. We expressed disease incidence as cases per million inhabitants per specified timeframe (2022 and 2023) and case–fatality rate as the total number of deaths per 1000 measles cases. All the reported cases (including endemic cases, imported cases, importation-related cases, and cases with unknown importation status) were included when calculating incidences. Percentages were rounded to the nearest whole number.

## 3. Results

### 3.1. Measles

#### 3.1.1. Incidence—Notifications and Laboratory Data

**Figure 1:**
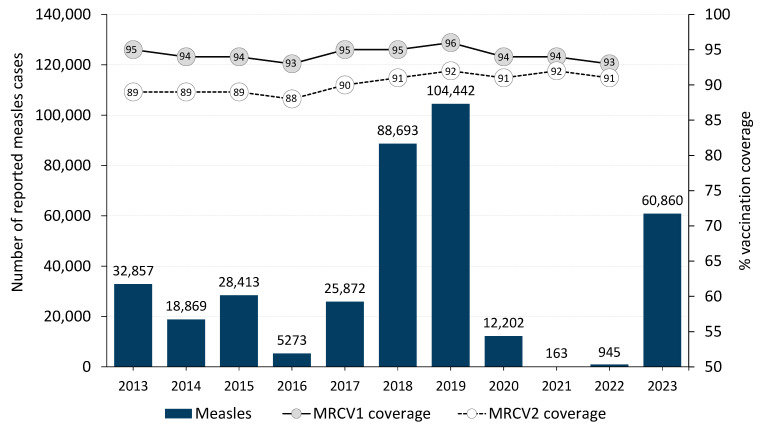
Reported measles cases and MRCV1 and MRCV2 coverage by year, WHO European Region, 2013–2023. MRCV1: first dose of measles- and rubella-containing vaccine; MRCV2: second dose of measles- and rubella-containing vaccine.

Except for 2018 and 2019, there was an overall declining trend in measles cases in the WHO European Region from 2013 to 2021, when the number of measles cases was the lowest ever recorded. Thereafter, the number of cases increased significantly in 2022 and 2023 (Figure 1).

In 2023, 60,860 measles cases were reported in 41 countries compared to 945 measles cases for 2022 reported in 27 countries (Table 1). Of the total cases for 2023, 31,173 cases (51%) were reported by the nine countries that, as concluded by the RVC, were considered endemic for 2022, and 1477 cases (2%) by two countries that were considered to have re-established endemicity in 2022. A total of 13,129 cases (22%) were reported by eight countries that as of the end of 2022 had interrupted measles transmission for at least the previous 12 months. A total of 15,063 cases (25%) were reported by the 33 countries considered as previously having eliminated measles. The remaining 18 cases were reported by one country whose review was pending.

Most cases in 2023 (95%; n = 57,584) were reported by six countries: Azerbaijan (13,735; 23%), Kazakhstan (15,111; 25%), Kyrgyzstan (7463; 12%), Romania (3419; 6%), the Russian Federation (12,872; 21%), and Türkiye (4984; 8%). The remaining 3276 cases (5%) were reported by 35 countries. The highest incidence per million population for 2023 was in Azerbaijan (1319) followed by Kyrgyzstan (1108).

**Figure 2:**
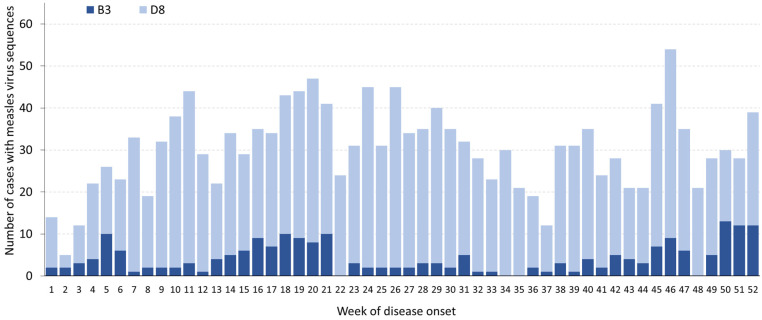
Number of cases with measles virus sequences in the WHO European Region reported to MeaNS by the genotype and week of disease onset, 2023 (as of 29 April 2024) *. * For 166 cases, the date of disease onset was missing. They were categorized by the week of specimen collection or the week when the clinical specimen was received at the laboratory, according to which date came first.

Of the total cases for 2023, 41,209 (68%) were laboratory-confirmed and 10,088 (17%) were epidemiologically linked to other measles cases. The remaining 9563 cases (16%) were classified as clinically compatible. In 2023, 32 (78%) of the countries reporting measles cases submitted genomic sequence information for 1578 cases (as of 29 April 2024) to the Measles Nucleotide Surveillance database (MeaNS) [9] through WHO-accredited laboratories. The genotypes identified in the Region were largely dominated by D8 variants (n = 1357) and the remainder were B3 variants (221). As in recent years, no other genotype was reported. The reported named strains for the D8 genotype were MVs/Rudaki.TJK/49.21 (n = 444), MVs/Patan.IND/16.19 (331), MVs/Victoria.AUS/6.11 (29), and (MVs/Frankfurt Main.DEU/17.11 (12). The other dominant D8 variants that are still unnamed include MeaNS Distinct Sequence Identifier 8350, which was reported in 123 cases. For the B3 genotype, MVs/Quetta.PAK/44.20 accounted for the majority of the B3 sequences (n = 132). The other named strains of the B3 genotype were MVs/Alburaimi.OMN/15.20 (n = 1) and MVs/Ohio.USA/37.22 (1). Of the 1578 cases that had sequences reported, 1412 cases (89%) had information on the date of disease onset (Figure 2).

#### 3.1.2. Age Distribution

**Figure 3:**
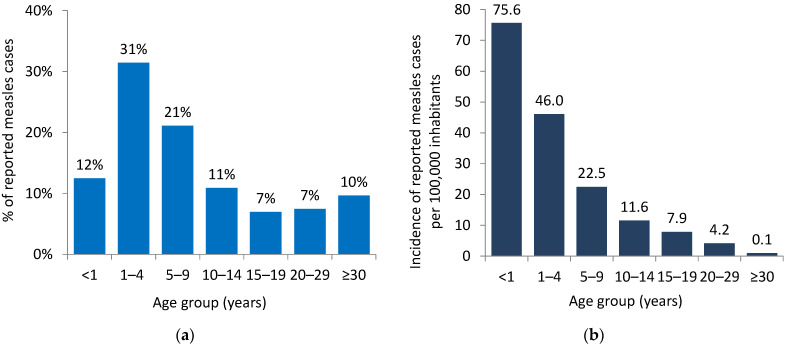
Age distribution of measles cases by proportion (**a**) and incidence per 100,000 inhabitants (**b**) in the WHO European Region, 2023 (n = 60,848) *. * For 12 cases, the age group was not reported. These are excluded from the graph.

**Figure 4:**
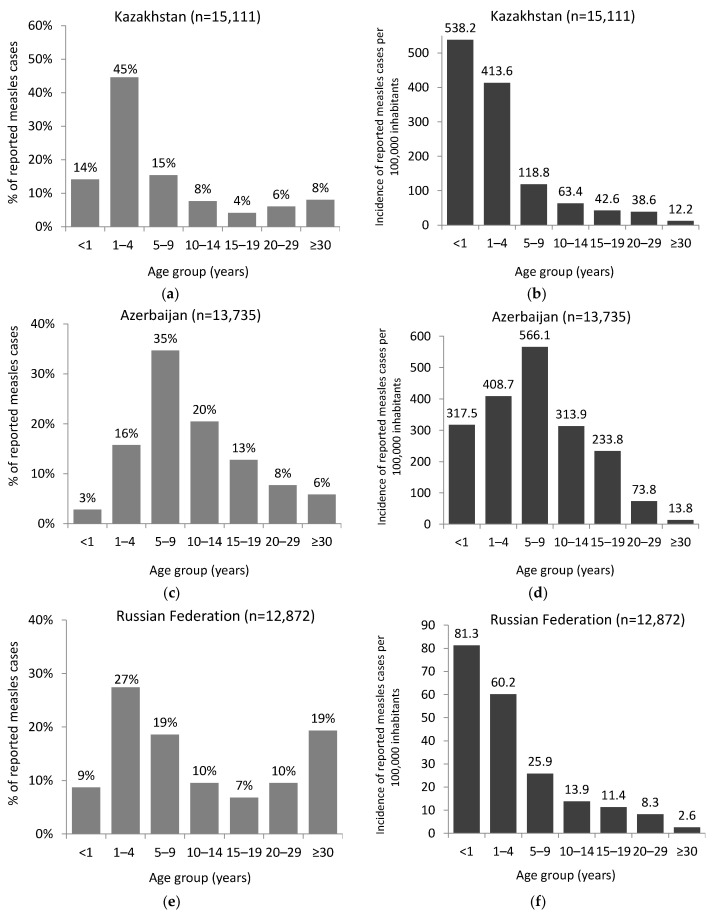

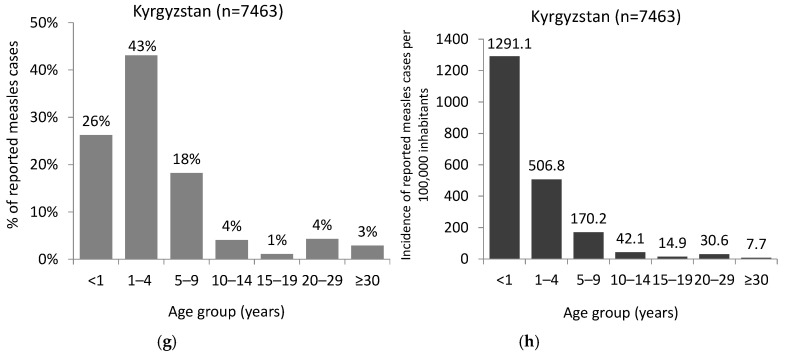

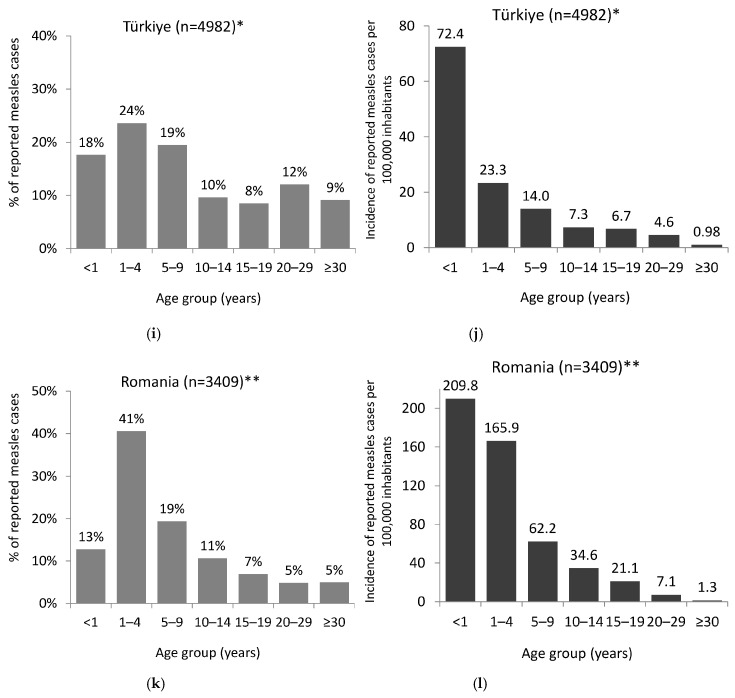
Age distribution of measles cases by proportion (**a**,**c**,**e**,**g**,**i**,**k**) and incidence per 100,000 inhabitants (**b**,**d**,**f**,**h**,**j**,**l**) in the six countries with the largest numbers of cases in the WHO European Region for 2023. * For 2 cases, the age group was not reported. These are excluded from the graph. ** For 10 cases, the age group was not reported. These are excluded from the graph.

Of the total cases for 2023, age was known for 60,848 cases: 7597 (12%) were <1 year old, 19,137 (31%) were 1–4 years old, 12,838 (21%) were 5–9 years old, 10,864 (18%) were 10–19 years old, and 10,412 (17%) were 20 years and older (Figure 3). Figure 4 shows the age distribution of measles cases in the six countries reporting the highest numbers of cases in the Region: Azerbaijan, Kazakhstan, Kyrgyzstan, Romania, the Russian Federation, and Türkiye by proportion and age-specific incidence. Among these six countries, the largest proportion of cases in children 1–4 years old was reported in Kazakhstan (45%; n = 6738) followed by Kyrgyzstan (43%; 3216). The largest proportion of cases in adults aged 20 years and older was reported in the Russian Federation (29%; 3717) and Türkiye (21%; 1057).

#### 3.1.3. Vaccination Status

Of the total cases for 2023, the vaccination status was known for 47,413 cases (78%). Of these, 39,045 cases (64%) were unvaccinated: 6804 (17%) were <1 year old, 14,652 (38%) were 1–4 years old, 7945 (20%) were 5–9 years old, 5574 (14%) were 10–19 years old, and 4062 (10%) were 20 years and older, and for 8 cases the age was unknown. The remaining 8368 cases (14%) were reportedly vaccinated with at least one dose of a measles-containing vaccine. The number of reported measles cases by vaccination status and age group is shown in Table 2.

#### 3.1.4. Measles-Rrelated Deaths

In 2023, there were 13 reported measles-related deaths in three countries: Kyrgyzstan (9 deaths), Romania (3), and Armenia (1). This corresponds to a case–fatality rate per 1000 measles cases of 0.21. Two fatal cases were reported in infants: one in a 5-month-old and another in a 6-month-old. Eight cases were in the 1–4-year-old age group, one case occurred in a 12-year-old, one case was in an 18-year-old, and another case was in a 35-year-old. Five fatal cases were laboratory-confirmed, five cases were epidemiologically linked to another case of measles, and three cases were classified as clinically compatible. Eleven fatal cases were unvaccinated, and one case was reportedly vaccinated with two doses of a measles-containing vaccine. In the remaining case, the vaccination status was unknown.

#### 3.1.5. Hospitalization

For 2023, data on hospitalization status were available for 52,259 cases (86%). Of these, 33,505 cases (64%) were hospitalized. The largest proportion of hospitalized cases were reported from Kazakhstan (39%; n = 13,149) followed by the Russian Federation (21%; 7104) and Kyrgyzstan (18%, 6133). In Kazakhstan, where the hospitalization status was known in all the reported cases (n = 15,111), 87% (13,149) were hospitalized.

#### 3.1.6. Imported Cases

For 2023, the status of measles virus importation was known for 50% (n = 30,608) of the cases. Of these, 659 were reported as imported cases, amounting to 2.2% of the cases with a known importation status. The largest proportion of imported cases by country was reported by the Russian Federation (44%; n = 292) followed by Türkiye (10%; 66) and Azerbaijan (9%; 57).

### 3.2. Rubella

#### 3.2.1. Incidence—Notifications and Laboratory Data

**Figure 5:**
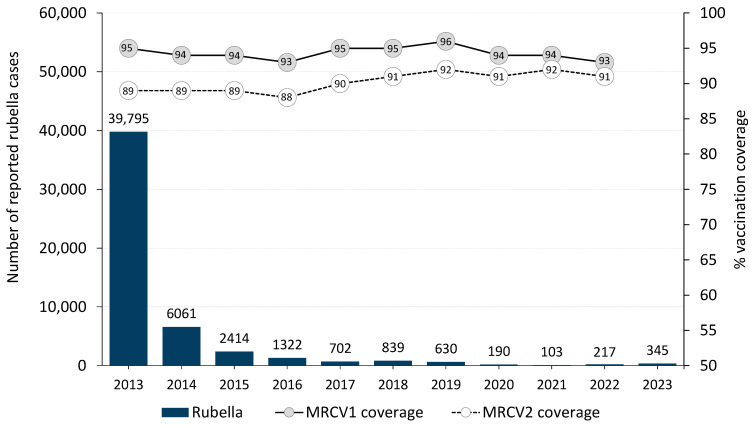
Reported rubella cases and MRCV1 and MRCV2 coverage by year, WHO European Region, 2013–2023. MRCV1: first dose of measles- and rubella-containing vaccine; MRCV2: second dose of measles- and rubella-containing vaccine.

There has been an overall steady declining trend in rubella cases in the WHO European Region from 2013 to 2021, when the number of rubella cases was the lowest ever recorded. Thereafter, the number of cases increased in 2022 and 2023 (Figure 5). In 2023, 345 rubella cases were reported by 17 (33%) of the 51 countries that submitted rubella data (including zero reporting) (Table 3). Most (76%; n = 263) of the cases were reported by Poland followed by Türkiye (6%; 20), Kyrgyzstan (3%; 11), and Ukraine (3%; 11).

Of the total cases for 2023, 262 (76%) were classified as clinically compatible. These were largely reported by Poland (n = 250, 95%). Seventy-nine cases (23%) were laboratory-confirmed and were mostly reported by Türkiye (20) followed by Kyrgyzstan (11), Poland (10), and Tajikistan (10). Four cases were classified as epidemiologically linked to another rubella case. In 2023, no rubella virus sequences were submitted to the Rubella Nucleotide Surveillance database (RubeNS) [10] (as of 29 April 2024).

#### 3.2.2. Age Distribution

**Figure 6:**
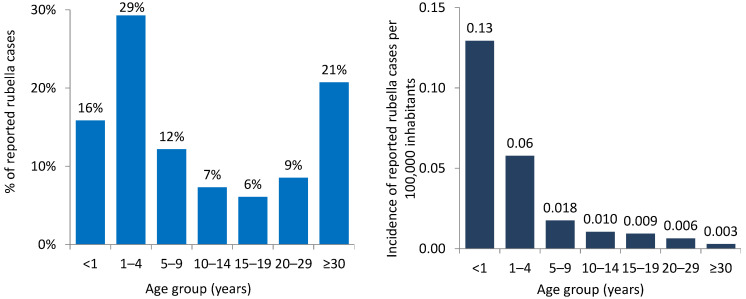
Age distribution of rubella cases by proportion (**left**) and incidence per 100,000 inhabitants (**right**) in the WHO European Region, 2023 (n = 82) *. * For 263 cases, the age group was not reported. These are excluded from the graph.

Of the total cases for 2023, age was known for 82 cases: 13 (16%) were <1 year old, 24 (29%) were 1–4 years old, 10 (12%) were 5–9 years old, 11 (13%) were 10–19 years old, and 24 (29%) were 20 years and older (Figure 6). Of the 24 cases 20 years and older, 18 cases were laboratory-confirmed. Of these, data on sex were known for 15 cases. Eight cases were males and seven cases were females of whom two were 20–29 years old and five were 30 years and older.

#### 3.2.3. Vaccination Status

Of the total cases for 2023, the vaccination status was known for 33 cases (10%). Of these, ten cases were unvaccinated: seven cases were <1 year old, two cases were 1–4 years old, and for one case the age was unknown. The remaining 23 cases were reportedly vaccinated with at least one dose of rubella-containing vaccine. The number of reported rubella cases by vaccination status and age is shown in Table 4.

#### 3.2.4. Imported Cases

The status of rubella virus importation was known in 54 (16%) rubella cases. Two were imported cases.

## 4. Discussion

### 4.1. Measles

The WHO European Region experienced a declining trend in measles cases from 2013 to 2022, except for the years 2018 and 2019, when cases spiked due to large outbreaks in just a few countries [16]. Cases for the Region as a whole began to increase again at the start of 2022, and this increasing trend has continued into 2024 [17]. The marked drop in reported measles cases in 2020 and 2021 is at least partly due to diminished virus transmission resulting from the non-pharmaceutical interventions, particularly lockdowns, implemented to contain the COVID-19 pandemic. These interventions were implemented throughout the Region starting in the spring of 2020 and continued throughout most of 2021. However, the under-reporting of cases was also very likely as fewer cases of measles could be detected due to disruptions in the usual disease surveillance activities and the diversion of resources to focus on COVID-19-related work. Furthermore, people were less likely to seek medical attention in healthcare facilities for mild illnesses during full or partial lockdowns resulting in mild cases of measles being undetected and thus not reported. It is plausible that the high coverage of measles-containing vaccines in the years preceding the pandemic also contributed to preventing large-scale measles outbreaks in the Region. Between 2016 and 2019, regional coverage with the first dose increased from 93% to 96%, while coverage with the second dose increased from 88% to 92%.

Following the evaluation of the submitted annual reports for 2022 from 52 of the 53 Member States of the Region, the RVC verified in 2023 that 33 countries sustained interruption for ≥36 months and are therefore considered to have eliminated endemic measles. Furthermore, one country had interrupted endemic measles transmission for 24 months and 7 countries had interrupted endemic measles transmission for 12 months by the end of 2022 [13]. The overall declining trend observed from 2013 to 2021, together with the verification of elimination status in 33 countries by the end of 2022, indicates that progress has been made towards interrupting endemic transmission of measles viruses in the Region. Despite this progress, the number of reported measles cases in the Region for 2023 (n = 60,860) increased 64-fold compared with that reported for 2022 (945). The number of cases for 2023 is probably even higher based on preliminary data reported in WIISE, as the delayed reporting of cases often occurs in countries with large-scale outbreaks. The number of countries reporting measles also increased from 27 in 2022 to 41 in 2023. The marked increase in the incidence of measles in many countries in 2023 compared to 2022 threatens the progress made so far and puts the Region at risk of delaying the achievement of the measles elimination goal.

Several countries have experienced challenges in achieving and sustaining optimal routine immunization coverage rates, particularly in 2020–2021, at the height of the COVID-19 pandemic, and in 2022. Among them, hesitancy to vaccinations and anti-vaccination sentiment has been reported to contribute to suboptimal vaccination rates in some countries [18,19]. Overall, coverage with the first MRCV dose for the Region decreased by two percentage points to 94% in 2020 compared with 2019 [1]. It remained at 94% in 2021 but decreased again by one percentage point to 93% in 2022. Coverage with the second dose showed less of a change in 2020–2022 and remained at 91–92%, similar to the coverage in 2018–2019.

Although coverage with the first MRCV dose may be considered as having declined only minimally at the regional level, at the national level, the decline in coverage varied widely. From 2019 to 2022, a decline in coverage, ranging between 1% and 62%, was observed in 28 countries for the first MRCV dose and between 1% and 23% for the second MRCV dose in 28 countries. Although the recommended ages for the first and second doses of MRCV vary between countries [1], it is probably the decline in first-dose coverage that had the largest impact on the observed increase in measles cases since 2022. This decline implies a widening of immunity gaps in the population large enough to allow the emergence of outbreaks, with over a third (31%) of the measles cases in 2023 occurring among children aged 1–4 years. Children in this age group would have been mostly affected by the observed decline in routine immunization coverage rates particularly in 2020–2021. In addition, the persistence of cases among adults aged 20 years and older (17%) suggests lingering immunity gaps in the adult population. Modeling studies have shown that to ensure measles elimination, 95% immunity would have to be achieved by the age of five years and that it is important not to have immunity gaps in older age groups [20].

The data we present for hospitalization due to measles should be interpreted with caution in the absence of a definition of hospitalization. Although hospitalization generally implies inpatient admission to a hospital, the term could also be interpreted to include outpatient ambulatory care at a healthcare facility. Across the Region, there is a diversity in the national regulations on the hospitalization of patients with measles. The high rate of hospitalized cases in the Region for 2023 was mostly caused by the large number of cases reported from Kazakhstan, Kyrgyzstan, and the Russian Federation, where the hospital admission of measles cases is a legal requirement. Further studies will be required for a better understanding of the number of cases with serious disease and to describe the burden of measles on hospitals.

The reported number of deaths is probably an underestimate and is therefore unlikely to indicate the true extent of mortality from measles. Surveillance systems do not necessarily collect outcome data systematically and measles might not be recorded on death certificates. Indeed, death certification errors are well recognized [21,22]. Moreover, death from complications, such as subacute sclerosing panencephalitis, can occur long after disease onset [23]. This means that the death rate per 1000 cases for 2023 is likely to be even higher than 0.21.

### 4.2. Rubella

The progress made towards rubella elimination is encouraging. The steady decline in the number of rubella cases and of countries reporting the disease suggests that the Region is close to eliminating rubella. The RVC verified in 2023 that 49 countries had interrupted endemic rubella transmission by the end of 2022 [13]. Thirty-three countries have achieved the elimination of both measles and rubella. Despite the observed increase in rubella cases in 2023, the low number of cases reported from the Member States of the Region at least since 2017 and low number of congenital rubella syndrome cases (45 cases for 2017–2022) [1] suggest that rubella elimination is an imminently achievable goal for the Region. Indeed, only four countries in the Region have not yet been verified as having eliminated rubella. Bosnia and Herzegovina, Poland, and Ukraine have not yet produced adequate documentation, including information on surveillance performance, to satisfy the RVC criteria to verify the absence of ongoing rubella transmission, while the submitted data from Israel are pending review. The next step to attaining the rubella elimination goal for the Region is to retrospectively review these remaining countries’ surveillance and vaccination data and any other alternative and supporting data in depth. The analysis of these data may also be coupled with an overall epidemiological assessment of rubella transmission in the Region as a whole.

The number of reported rubella cases in the Region increased for 2023 compared with 2022, with 128 more cases, a rise of 59%. The number of countries reporting rubella also increased between 2022 and 2023 from 10 to 17. These increases should be interpreted in light of the concurrent measles outbreaks in many countries since more clinical specimens from suspected measles cases that test negative would be tested for rubella, as is required. In both years, fewer countries reported rubella cases (including zero reporting) than measles cases.

As in previous years, most (76%) reported rubella cases for 2023 continue to be classified as clinically compatible, with Poland reporting 95% of such cases in the Region. With such a high proportion of clinically compatible cases, an accurate epidemiological assessment to identify the true incidence of rubella in the country cannot be made. Countries with a high number of suspected rubella cases need to make efforts to increase specimen collection to allow laboratory testing. Without laboratory confirmatory tests for rubella, other rash-producing viral infections such as those caused by human herpes viruses and parvovirus B19 are easily misdiagnosed as rubella. Nevertheless, the observed rare occurrence of the laboratory-confirmed cases of rubella among females of childbearing age signals a persisting potential risk for congenital rubella syndrome (CRS) and underlines the important role of rubella vaccination to prevent CRS.

### 4.3. Limitations of Surveillance Data

We did not evaluate the surveillance systems for measles and rubella and the extent of the implementation of case definitions used for reporting these diseases by each country. Not all countries may conduct disease surveillance with the needed sensitivity appropriate for diseases targeted for elimination. Therefore, our evaluation could only permit the identification of regional and country-specific trends over time. Moreover, the under-reporting of cases and incompleteness of data reporting are notorious with routine communicable disease surveillance systems based on passive reporting. Consequently, making accurate comparisons between countries may be challenging. Despite these limitations, the surveillance data we presented are the best possible data that national surveillance agencies could provide and are likely to be at least consistent over time, reflecting the general trends in each country. Differences in the quality of surveillance systems between countries are not expected to affect the observed general trends for both diseases.

### 4.4. Measles and Rubella Elimination Strategies

The elimination of both measles and rubella remains a priority goal for all the countries of the WHO European Region. Countries are urged to review their vaccination programs, surveillance systems, and outbreak response capacities and adhere to the core strategies for achieving this goal. These strategies are reiterated in the recently published integrated guidance on surveillance, outbreak response, and the verification of elimination [14] and are discussed under the headings below.

#### 4.4.1. Achieving and Sustaining High Vaccination Coverage

High vaccination coverage of at least 95% with two doses of MRCVs administered through high-quality routine immunization services is needed to prevent outbreaks and attain the measles and rubella elimination goal. Tailored approaches to achieve high coverage at the subnational level and in every community are essential to prevent the accumulation of susceptible individuals even within countries and areas that otherwise report high overall coverage rates.

Countries are urged to closely monitor vaccination coverage, preferably using electronic registers, to identify disparities at the local level and among various population groups that could point to inequities in immunization service delivery. Measles outbreaks have often affected ethnic, religious, and philosophical population groups [24] with low vaccination coverage. Therefore, countries should implement tailored approaches to achieve equitable immunization coverage across all geographic and demographic segments. To inform such approaches, behavioral insights research may be conducted to improve the understanding of whether low coverage in a particular area or community is related to factors such as the lack of convenient access to immunization services, lack of awareness, or loss of public confidence in the benefits of vaccines. The WHO Regional Office for Europe has just producedguidance for countries on how to identify and address inequities [25].

Where disruption in routine immunization service delivery has occurred for any length of time, such as at the height of the COVID-19 pandemic [26], countries are urged to undertake tailored immunization catch-up campaigns to close resulting population immunity gaps not only among children but also adults. This requires identifying the birth cohorts and any special populations or risk groups that have missed their vaccine doses and offering them the opportunity to be vaccinated. The WHO Regional Office for Europe published guidance to support countries in planning and implementing catch-up immunization using a structured algorithm [27].

#### 4.4.2. Improving the Availability and Use of High-Quality, Evidence-based Information on Immunization

To enhance acceptance and demand for immunization, health authorities are urged to improve the availability and use of high-quality, evidence-based information for healthcare professionals and the public on the benefits and risks associated with immunization. Indeed, pre- and in-service training of the health workforce on vaccines and the diseases they prevent is essential if they are to confidently recommend and communicate about vaccination. The WHO Regional Office for Europe has developed various training packages [28,29,30] for health workers to increase their knowledge about vaccines and help them answer questions from their clients and patients, as well as guidance for health authorities to ensure the timely and effective investigation of any adverse events following vaccination and the transparent communication of the results to sustain public trust [31].

Health literacy can be strengthened from a young age to promote positive health-seeking behavior throughout a person’s life. Adding health topics to school curricula has been demonstrated to improve students’ knowledge on these topics [32,33], with positive spillover effects benefiting caregivers and communities alike [34]. Health education that includes information on vaccines and the diseases they prevent can create positive attitudes toward vaccines, thereby increasing children’s resilience to misinformation, empowering them to make informed health-related choices related to vaccination [35]. The possibility of increasing vaccination coverage among adolescents using a website-based educational program with tailored information has been described [36]. To increase health literacy among 10–12-year-olds, the WHO Regional Office for Europe has developed a game-based learning platform to teach them about vaccines, the immune system, and online source criticism [37]. This educational tool called “Immune Patrol” is currently being rolled out in schools in several countries in the Region.

Joint initiatives such as European Immunization Week (EIW) [38], which takes place every April, provide national authorities, international partners, and civil society organizations a platform through which to raise awareness and engagement at the local level. From social media campaigns to national conferences, lectures, talk shows, interviews, and public events, many channels and opportunities are used to make accurate information about the role of vaccines in protecting health available and accessible. Since its inception in 2007, EIW has helped keep the measles and rubella elimination goals, and the broader implementation of EVAP and now EIA2030, high on the political agenda. At the same time, it serves to promote innovation, highlight challenges, and encourage actions needed to achieve measles and rubella elimination and other immunization targets and goals.

#### 4.4.3. Strengthening Surveillance

Surveillance systems for measles and rubella need to be sensitive and specific enough to detect, confirm, and classify all suspected cases. This involves rigorous case investigation and the laboratory confirmation of suspected sporadic cases and outbreaks. To confirm the occurrence or absence of disease, efforts need to be made to increase specimen collection to allow the laboratory testing of suspected measles and rubella cases, including virus characterization. Supported with epidemiological information, this will facilitate the monitoring of regional patterns of circulation and the tracking of transmission pathways, and will help distinguish between new importations and ongoing disease transmission, as well as the different chains of transmission. Countries are also reminded to report their sequence data to the WHO global sequence databases (MeaNs and RubeNS) to allow the in-depth analyses of the molecular epidemiology of measles and rubella viruses.

In 2023, the importation status of measles cases in the Region was reported in only 50% of the cases and the importation status of rubella cases in only 16% of the cases. This low proportion of cases with known importation status poses challenges for determining whether prolonged periods of virus transmission were the result of prolonged uninterrupted transmission chains or multiple unrelated importations, with important potential implications for the verification of elimination status. The WHO Regional Office for Europe has recently published updated guidance on how to conduct elimination-standard surveillance [14].

#### 4.4.4. Ensuring Adequate Outbreak Preparedness and Response

As the Region works towards attaining elimination, timely outbreak investigation and response become crucial. Timely response to outbreaks is indeed a core strategy for measles and rubella elimination and is also embedded in EIA2030. Not all countries in the Region have responded timeously and adequately to measles outbreaks, allowing prolonged virus transmission and the associated increase in morbidity and hospitalization, burdening healthcare systems and increasing healthcare costs [39].

An adequate outbreak response requires a thorough field investigation including enhanced laboratory surveillance, active case finding, and contact tracing for the vaccination of any susceptible individuals. The most important immediate response measure after the detection of measles cases is to identify and provide vaccination to the susceptible individuals and population groups at risk of exposure. Rapid immunization efforts in an outbreak setting are aimed at interrupting disease transmission without delay in order to reduce the extent and duration of outbreaks and associated morbidity and mortality. Achieving sufficient vaccination coverage to prevent an outbreak and convincing those at risk to be vaccinated during an outbreak require tailored efforts to sustain or gain public trust in health authorities and vaccination.

Health authorities are encouraged to collect comprehensive and timely surveillance data using sophisticated technologies, if feasible, such as real-time data collection and analysis tools. Data generated from thorough outbreak investigations should be used to identify and address inequities in vaccine service delivery. Indeed, the root cause analysis of outbreaks should be conducted to better inform improvements in programmatic interventions to prevent future outbreaks [40].

Nosocomial transmission of the virus contributes to measles outbreaks. Apart from the risk of the amplification of outbreaks by virus transmission between patients, and between healthcare workers and patients in healthcare settings [41], increased mortality can result from serious complications in hospitalized infants and adults who may already be weakened by other medical conditions. For 2023, the relatively large proportion (64%) of measles cases that were hospitalized highlights the importance of ensuring that all the necessary precautions are taken to prevent nosocomial transmission by implementing effective infection control practices.

Apart from having national plans of action for the elimination of measles and rubella in place and regularly updated, every country should have a national outbreak preparedness and response plan that particularly addresses measles outbreaks. WHO guidance for the early detection of outbreaks describes how to conduct a rapid and appropriate response [14] and can be used to structure response plans.

## 5. Conclusions

The resurgence of measles in the Region reflects ongoing challenges in endemic countries and countries experiencing large-scale outbreaks that may slow progress to achieve the elimination goal in the Region. It also reflects the need for sustained efforts to protect gains in countries that are considered to have interrupted or eliminated endemic measles. At the same time, efforts made to eliminate rubella need to be sustained. The high cost of these diseases in terms of morbidity, hospitalizations, and mortality continues to undeniably justify the efforts to eliminate these diseases in the Region.

To attain the elimination goal for the Region, all the countries need to ensure that the immunization coverage of at least 95% is reached and maintained in all districts, and immunity gaps in the population are identified and closed. Furthermore, high-quality surveillance is necessary to monitor disease occurrence and detect outbreaks so that appropriate and timely response measures can be taken. Accurate surveillance data are also needed to adequately ascertain the status of measles and rubella transmission as part of the elimination verification process. The goal will not be met unless all the Member States of the Region translate their political commitment into adequate investment and concrete programmatic actions that address identified challenges.

## Figures and Tables

**Table 1:** Incidence of measles per million population in the WHO European Region, 2022 and 2023.

Country	Number of Measles Cases		Incidence per Million Inhabitants *		Number of Measles Cases 2023			
	2022	2023	2022	2023	Laboratory-confirmed	Epidemiologically Linked	Clinically Compatible	Imported
Albania	1	3	0.4	1.1	3	0	0	0
Andorra	0	0	0	0	0	0	0	-
Armenia	0	556	0	200.1	547	9	0	38
Austria	1	186	0.1	20.8	186	0	0	8
Azerbaijan	1	13,735	0.1	1319.1	1429	5466	6840	57
Belarus	0	188	0	19.8	188	0	0	0
Belgium	17	68	1.5	5.8	52	12	4	-
Bosnia and Herzegovina	6	10	1.9	3.1	8	0	2	0
Bulgaria	1	0	0.1	0	0	0	0	-
Croatia	0	3	0	0.7	3	0	0	3
Cyprus	0	0	0	0	0	0	0	-
Czechia	0	1	0	0.1	1	0	0	0
Denmark	0	9	0	1.5	5	4	0	3
Estonia	0	4	0	3.0	4	0	0	4
Finland	1	1	0.2	0.2	1	0	0	1
France	19	117	0.3	1.8	102	7	8	31
Georgia	12	39	3.2	10.5	37	0	2	13
Germany	15	83	0.2	1.0	75	3	5	30
Greece	0	0	0	0	0	0	0	-
Hungary	0	1	0	0.1	1	0	0	1
Iceland	0	0	0	0	0	0	0	-
Ireland	2	4	0.4	0.8	1	3	0	2
Israel	0	18	0	2.0	15	1	2	3
Italy	15	44	0.3	0.7	39	1	4	17
Kazakhstan	4	15,111	0.2	770.7	14,087	688	336	-
Kyrgyzstan	23	7463	3.5	1108.0	2023	3373	2067	4
Latvia	0	1	0	0.5	1	0	0	1
Lithuania	0	4	0	1.5	4	0	0	1
Luxembourg	0	0	0	0	0	0	0	-
Malta	0	0	0	0	0	0	0	-
Monaco	0	0	0	0	0	0	0	-
Montenegro	0	0	0	0	0	0	0	-
Netherlands (Kingdom of the)	6	7	0.3	0.4	6	1	0	3
North Macedonia	0	1	0	0.5	1	0	0	0
Norway	1	2	0.2	0.4	2	0	0	1
Poland	41	29	1.0	0.7	16	0	13	1
Portugal	0	1	0	0.1	0	0	1	0
Republic of Moldova	0	3	0	0.9	3	0	0	2
Romania	9	3419	0.5	171.9	3018	281	120	17
Russian Federation	117	12,872	0.8	89.1	12,570	189	113	292
San Marino	0	0	0	0	0	0	0	-
Serbia	0	52	0	7.3	47	1	4	0
Slovakia	0	6	0	1.0	6	0	0	2
Slovenia	0	0	0	0	0	0	0	-
Spain	1	13	0.02	0.3	10	0	3	6
Sweden	4	11	0.4	1.0	11	0	0	6
Switzerland	1	37	0.1	4.2	34	3	0	13
Tajikistan	451	294	45.3	29.0	250	44	0	0
Türkiye	127	4984	1.5	58.1	4969	2	13	66
Turkmenistan	0	0	0	0	0	0	0	-
Ukraine	11	66	0.3	1.8	57	0	9	0
United Kingdom	50	231	0.7	3.4	231	0	0	30
Uzbekistan	8	1183	0.2	33.6	1166	0	17	3
WHO European Region	945	60,860	1.0	65.3	41,209	10,088	9563	659

* based on estimated 2022 and 2023 population data, respectively. There may be differences in the numbers documented in the reports based on the data derived from the WHO/UNICEF Joint Reporting Form—the data we present for 2022 include updated numbers reported by some countries and for 2023, the data may be updated following the publication of this article. Discrepancies might also arise with nationally reported data if these include cases by the year of notification rather than the year of disease onset.

**Table 2:** Measles cases by vaccination status and age group in the WHO European Region, 2023 (n = 60,860).

	<1 Years		1–4 Years		5–9 Years		10–14 Years		15–19 Years		20–29 Years		≥30 Years		Unknown Age		Total	
Unvaccinated	6804	90%	14,652	77%	7945	62%	3515	53%	2059	49%	1848	41%	2214	38%	8	67%	39,045	64%
Vaccinated with a single dose	88	1%	2220	12%	1288	10%	621	9%	300	7%	204	4%	221	4%	1	8%	4943	8%
Vaccinated with at least two doses	6	0%	148	1%	917	7%	881	13%	591	14%	441	10%	440	7%	1	8%	3425	6%
Unknown status	699	9%	2117	11%	2688	21%	1609	24%	1288	30%	2051	45%	2993	51%	2	17%	13,447	22%
Total	7597	100%	19,137	100%	12,838	100%	6626	100%	4238	100%	4544	100%	5868	100%	12	100%	60,860	100%

**Table 3:** Incidence of rubella per million population in the WHO European Region, 2022 and 2023.

Country	Number of Rubella Cases		Incidence per Million Inhabitants *		Number of Rubella Cases 2023			
	2022	2023	2022	2023	Laboratory-confirmed	Epidemiologically Linked	Clinically Compatible	Imported
Albania	0	1	0	0.4	1	0	0	0
Andorra	0	0	0	0	0	0	0	-
Armenia	0	0	0	0	0	0	0	-
Austria	0	0	0	0	0	0	0	-
Azerbaijan	4	0	0.4	0	0	0	0	-
Belarus	-	0	-	0	0	0	0	-
Belgium ^1^	-	-	-	-	-	-	-	-
Bosnia and Herzegovina	0	2	0	0.6	1	0	1	-
Bulgaria	0	0	0	0	0	0	0	-
Croatia	0	0	0	0	0	0	0	-
Cyprus	0	0	0	0	0	0	0	-
Czechia	0	0	0	0	0	0	0	-
Denmark ^2^	-	-	-	-	-	-	-	-
Estonia	0	0	0	0	0	0	0	-
Finland	1	1	0.2	0.2	1	0	0	0
France	0	0	0	0	0	0	0	-
Georgia	0	0	0	0	0	0	0	-
Germany	8	8	0.1	0.1	5	0	3	0
Greece	0	0	0	0	0	0	0	-
Hungary	0	0	0	0	0	0	0	-
Iceland	0	0	0	0	0	0	0	-
Ireland	0	0	0	0	0	0	0	-
Israel	0	1	0	0.1	1	0	0	0
Italy	4	2	0.1	0.03	1	0	1	0
Kazakhstan	0	7	0	0.4	6	1	0	-
Kyrgyzstan	2	11	0.3	1.6	11	0	0	1
Latvia	1	0	0.5	0	0	0	0	-
Lithuania	1	1	0.4	0.4	1	0	0	0
Luxembourg	0	0	0	0	0	0	0	-
Malta	0	0	0	0	0	0	0	-
Monaco	0	0	0	0	0	0	0	-
Montenegro	0	0	0	0	0	0	0	-
Netherlands (Kingdom of the)	0	0	0	0	0	0	0	-
North Macedonia	0	0	0	0	0	0	0	-
Norway	0	0	0	0	0	0	0	-
Poland	147	263	3.7	6.4	10	3	250	-
Portugal	0	0	0	0	0	0	0	-
Republic of Moldova	0	0	0	0	0	0	0	-
Romania	0	1	0	0.1	1	0	0	1
Russian Federation	0	3	0	0.02	3	0	0	0
San Marino	0	0	0	0	0	0	0	-
Serbia	-	0	-	0	0	0	0	-
Slovakia	0	0	0	0	0	0	0	-
Slovenia	0	0	0	0	0	0	0	-
Spain	0	0	0	0	0	0	0	-
Sweden	0	0	0	0	0	0	0	-
Switzerland	0	1	0	0.1	0	0	1	0
Tajikistan	0	10	0	1.0	10	0	0	0
Türkiye	40	20	0.5	0.2	20	0	0	0
Turkmenistan	0	0	0	0	0	0	0	-
Ukraine	9	11	0.2	0.3	5	0	6	0
United Kingdom	0	0	0	0	0	0	0	-
Uzbekistan	0	2	0	0.1	2	0	0	0
WHO European Region	217	345	0.2	0.4	79	4	262	2

* based on estimated 2022 and 2023 population data, respectively. ^1^ country does not have a comprehensive rubella surveillance system. ^2^ comprehensive rubella surveillance system for all ages became effective on 1 November 2023. There may be differences in the numbers documented in the reports based on the data derived from the WHO/UNICEF Joint Reporting Form—the data we present for 2022 include updated numbers reported by some countries and for 2023, the data may be updated following the publication of this article. Discrepancies might also arise with nationally reported data if these include cases by the year of notification rather than the year of disease onset.

**Table 4:** Rubella cases by vaccination status and age group in the WHO European Region, 2023 (n = 345).

	<1 Years		1–4 Years		5–9 Years		10–14 Years		15–19 Years		20–29 Years		≥30 Years		Unknown Age		Total	
Unvaccinated	7	54%	2	8%	0	0%	0	0%	0	0%	0	0%	1	6%	0	0%	10	3%
Vaccinated with a single dose	0	0%	13	54%	2	20%	0	0%	0	0%	0	0%	1	6%	0	0%	16	5%
Vaccinated with at least two doses	0	0%	1	4%	3	30%	2	33%	0	0%	1	14%	0	0%	0	0%	7	2%
Unknown status	6	46%	8	33%	5	50%	4	67%	5	100%	6	86%	15	88%	263	100%	312	90%
Total	13	100%	24	100%	10	100%	6	100%	5	100%	7	100%	17	100%	263	100%	345	100%

## Data Availability

Data sources supporting the results have already been indicated by the references.
